# Single-Cell RNA sequencing of leaf sheath cells reveals the mechanism of rice resistance to brown planthopper (*Nilaparvata lugens*)

**DOI:** 10.3389/fpls.2023.1200014

**Published:** 2023-06-19

**Authors:** Wenjun Zha, Changyan Li, Yan Wu, Junxiao Chen, Sanhe Li, Minshan Sun, Bian Wu, Shaojie Shi, Kai Liu, Huashan Xu, Peide Li, Kai Liu, Guocai Yang, Zhijun Chen, Deze Xu, Lei Zhou, Aiqing You

**Affiliations:** ^1^ Key Laboratory of Crop Molecular Breeding, Ministry of Agriculture and Rural Affairs, Hubei Key Laboratory of Food Crop Germplasm and Genetic Improvement, Food Crops Institute, Hubei Academy of Agricultural Sciences, Wuhan, China; ^2^ Hubei Hongshan Laboratory, Wuhan, China; ^3^ Henan Assist Research Biotechnology Co., Ltd., Zhengzhou, China

**Keywords:** single-cell, RNA-seq, leaf sheath cells, resistant, *Nilaparvata lugens*, rice

## Abstract

The brown planthopper (BPH) (*Nilaparvata lugens*) sucks rice sap causing leaves to turn yellow and wither, often leading to reduced or zero yields. Rice co-evolved to resist damage by BPH. However, the molecular mechanisms, including the cells and tissues, involved in the resistance are still rarely reported. Single-cell sequencing technology allows us to analyze different cell types involved in BPH resistance. Here, using single-cell sequencing technology, we compared the response offered by the leaf sheaths of the susceptible (TN1) and resistant (YHY15) rice varieties to BPH (48 hours after infestation). We found that the 14,699 and 16,237 cells (identified *via* transcriptomics) in TN1 and YHY15 could be annotated using cell-specific marker genes into nine cell-type clusters. The two rice varieties showed significant differences in cell types (such as mestome sheath cells, guard cells, mesophyll cells, xylem cells, bulliform cells, and phloem cells) in the rice resistance mechanism to BPH. Further analysis revealed that although mesophyll, xylem, and phloem cells are involved in the BPH resistance response, the molecular mechanism used by each cell type is different. Mesophyll cell may regulate the expression of genes related to vanillin, capsaicin, and ROS production, phloem cell may regulate the cell wall extension related genes, and xylem cell may be involved in BPH resistance response by controlling the expression of chitin and pectin related genes. Thus, rice resistance to BPH is a complicated process involving multiple insect resistance factors. The results presented here will significantly promote the investigation of the molecular mechanisms underlying the resistance of rice to insects and accelerate the breeding of insect-resistant rice varieties.

## Introduction

Rice (*Oryza sativa* L.) is one of the most important cereal crops and has been domesticated for approximately 7,500 years (Zong et al., 2007). Stored and unharvested rice can be attacked by >800 species of insect pests (Ghaffar et al., 2011). The brown planthopper (BPH), *Nilaparvata lugens* (Stål), is one of the most economically important insects which can cause huge destruction of rice plants (Xue et al., 2014). BPH can damage rice growth by spreading plant viruses (such as rice grassy and rice-ragged stunt viruses) (Cabauatan et al., 2009) and sucking plant sap.

BPH has co-evolved to adapt strongly to its host, rice (Sezer and Butlin, 1998; Zheng et al., 2021). The widely used insecticides could effectively protect from the BPH and other pests-caused damages. However, overuse of insecticides not only promotes BPH obtaining the adaptability and resistance to insecticides but also causes serious environmental pollution (Senthil-Nathan et al., 2009). However, a complex defense system against BPH also exists in rice. Therefore, developing rice varieties resistant to insects using their insect-resistance genes could be an ideal complement and alternative to existing insect-control measures. Since the first BPH-resistant rice variety was discovered, >40 genes associated with BPH resistance (Akanksha et al., 2019; Li et al., 2019), such as *bph1-40*, have been identified. Some of these BPH-resistant genes, such as *bph1-4*, have been successfully used in breeding BPH-resistant rice varieties (Athwal et al., 1971; Laksminarayana and Khush, 1977). These BPH-resistance genes not only promote rice BPH-resistance but also decrease BPH reproduction and prolong the period of BPH development (Du et al., 2009; Senthil-Nathan et al., 2009; Nguyen et al., 2019). The interaction between BPH and rice is a complex and dynamic process. Currently, it is understood that BPH sucks phloem sap by inserting the stylet bundle with an accompanying salivary sheath into the plant (Spiller, 1990). On its way to the phloem, the mouth stylet bundle pierces through various cells, such as the epidermis, mesophyll, phloem, etc. Thus, multiple resistant mechanisms may interrupt the BPH feeding process at several cellular locations. Moreover, the different cells encountered by the stylet may mount different resistant functions depending on the rice variety. Presently, the identity of the cells involved in insect resistance in rice and the underlying molecular mechanisms remain unknown.

Single-cell RNA sequencing (scRNA-seq) provides a method to examine the expression of all genes in each cell at the transcriptional level (Brennecke et al., 2013; Islam et al., 2014). Recently, scRNA-seq was applied in many fields to explore the heterogeneity of cells (Luecken and Theis, 2019). In addition, studies on heterogeneity in animal cells have been abundantly reported (Butler et al., 2018; Aran et al., 2019; Zhang et al., 2019). However, because of the presence of plant cell walls, the application of scRNA-seq is limited in plants. Currently, several single-cell studies in plants focusing on tissue and cellular functional differentiation–such as differentiation of roots and stems in *Arabidopsis* (Zhang et al., 2021b), stomatal lineage and developing leaf (Lopez-Anido et al., 2021), ploidy-dependence in *Arabidopsis* female gametophytes (Song et al., 2020), leaf and root differentiation in rice (Liu Q. et al., 2021; Wang et al., 2021; Zhang et al., 2021a), differentiation of maize ears facilitates (Xu et al., 2021b), poplar xylem formation (Xie et al., 2022), *Nicotiana attenuate* corolla cells formation (Kang et al., 2022)–have been reported. However, studies on the molecular mechanisms underlying the response of different plant cells to biotic stresses are rarely reported.

Here, we used scRNA-seq to explore the differences in the molecular responses mounted by the various cell types to BPH biotic stress (to BPH infestation) in two rice varieties differing in their resistance to BPH. This study’s results will be a reference for future studies revealing the molecular mechanisms of rice insect resistance and provide a theoretical basis for better breeding of varieties resistant to BPH.

## Materials and methods

### Rice plants cultivation and infesting rice plants with brown planthopper

For this study, seedlings of the rice varieties TN1 (susceptible to BPH) and YHY15 (moderately resistant to BPH) were cultivated in a climate chamber under a 12-h light/12-h dark cycle at 30 ± 1°C and 70% relative humidity. Then, the 2- to 3-instar hopper nymphs were used to infest the seedlings at the third-leaf stage (13 or 14 days old) at a density of eight insects per seedling under the conditions of 70% relative humidity, 25 ± 1°C, and 16-h light/8-h dark cycle. And we covered the stems of each seedling with breathable plastic tubes to prevent the brown planthoppers from escaping. After 48 hours, the leaf sheaths, including herbivore-exposed local (damaged) parts of TN1 and YHY15, were collected for further study. Three seedlings were sampled for each rice variety.

### Rice protoplast isolation

The rice protoplast was isolated using the previous methods (Jabnoune et al., 2015; Wang et al., 2021). Briefly, the finely cut rice seedling leaf sheaths were immediately incubated in an enzymes solution containing 10 mM MES, 0.6 M mannitol, 0.75% macerozyme R-10, and 1.5% cellulase RS (pH 5.7) for 3 h (shaking at 70 rpm) at 28°C. After incubation, the enzyme solution was filtered out. The digested protoplasts were washed with the W5 solution (154 mM NaCl, 125 mM CaCl_2_, 5 mM KCl, and 2 mM MES at pH 5.7) and collected by centrifugation at 300 × g. The protoplasts were resuspended in a solution containing 0.6 M D-Mannitol, 15 mM MgCl_2_, and 4 mM MES (pH 5.7). A small amount of the single-cell suspension was added to an equal volume of 0.4% trypan blue dye. The concentration of viable cells was adjusted to the desired concentration (1000 to 2000 cells/µL) by counting the cells using Countess^®^ II Automated Cell Counter.

### Single-cell RNA sequencing

To generate the single-cell Gel Bead-In-Emulsions (GEMs) from cellular suspensions, a GemCode Single-cell instrument (10x Genomics, Pleasanton, CA, USA) was used. The Chromium Next GEM Single Cell 3’ Reagent Kits v3.1 (CG000183, RevC, 10x Genomics) was used to prepare the library and for sequencing. To construct the library, the barcoded, reverse-transcribed, and full-length cDNAs were amplified using PCR assay. Next, the PCR products were ligated to the adapters for sequencing, followed by PCR amplification according to the DNA template concentrations. Then a paired-end sequencing was conducted using the Illumina platform (Illumina Inc., San Diego, USA) for sequencing libraries.

### Preprocessing and cell clustering analysis

To generate counts quantification, alignment, and FASTQ files from raw Illumina BCL files, the Cell Ranger software (version 3.1.0, 10x Genomics) was used. (Lun et al., 2019). To align the sequencing, reads of rice scRNA-seq samples were aligned to the rice reference genome (Kawahara et al., 2013). The Seurat software (V3.1.1, Satija Lab, New York, USA) was used for the analysis of the downstream genes through importing the gene matrices cell for each sample individually (Butler et al., 2018; Stuart et al., 2019). Using DoubletFinder (v2.0.3), we filtered out the cells with doublet GEMs, no less than 8000 UMIs and no less than 10% mitochondrial genes (McGinnis et al., 2019). After normalizing the data, the harmony algorithm was used to correct the batch effect (Korsunsky et al., 2019). Principal component analysis dimensional reduction was applied as a reference according to the previous report (Chung and Storey, 2015). Cells clustering was analyzed using the Louvain method to maximize modularity (Rotta and Noack, 2011). The combination of cell type annotation with these previously reported cell type marker genes was conducted using the R packages SingleR (Aran et al., 2019) and Cellassign (Zhang et al., 2019).

### Differentially expressed genes analysis

The Wilcoxon rank sum test was used to compare difference in expression of every detected gene between the given cluster and other cells (Camp et al., 2017). We identified the significantly upregulated genes using the following criteria–genes had to be at least 1.28-fold overexpressed in a target cluster having >25% of same type of cells, and have a *p* value < 0.01. Subsequently, Kyoto Encyclopedia of Genes and Genomes (KEGG) pathways enrichment analyses and Gene Ontology (GO) functional annotation were conducted to analyze these DEGs (Boyle et al., 2004; Kanehisa and Goto, 2000; Kanehisa et al., 2008).

### Gene set variation analysis and cell cycle analysis

GSVA was performed using a collection of gene sets from Molecular Signatures Database (MSigDB) (Liberzon et al., 2015) to identify pathways and cellular processes enriched in different clusters. GSVA was performed as implemented in the GSVA R package version 1.26 (Hanzelmann et al., 2013) based on the cluster-averaged log-transformed expression matrix. According to the expression of the genes associated with G1/S phase (n = 100), S phase (n = 113), G2/M phase (n = 133), M phase (n = 106), and M/G1 phase (n = 106), the cell cycle score was assigned using the package “Seurat” (Macosko et al., 2015). Cells with the highest score <0.3 were defined as non-cycling cells (Neftel et al., 2019). In addition, the Plant Transcription Factor Database (PlantTFDB) was used in the transcript factors annotation (Jin et al., 2017).

### RNA *in situ* hybridization assay

The situ hybridization assay was conducted based on the previously published protocol (Umeda et al., 1999). Briefly, hydration and paraffin embedding of the fresh rice seedling leaf sheaths were performed after 12 h fixation with FAA solution (3.7% formaldehyde, 5% acetic acid, and 50% ethanol). Subsequently, the paraffin-embedded rice seedling leaf sheaths were sectioned into 10 µm thick sections and treated for 2 h at 62°C (KD-P, Zhejiang Jinhua Kedi Instrumental Equipment Co., Ltd, China) and xylene (twice for 15 min) to remove paraffin and serially rehydrated using different concentrations of ethanol. Then, the leaf sheath sections were hybridized with RNA probes following a 15-minute Proteinase K (20 µg/ml) (G1234, Nanjing Zoonbio Biotechnology, Ltd., China) digestion (at 37°C) and serial dehydration using different concentrations of ethanol. Next, the probes were transcribed *in vitro* using a Digoxigenin RNA labeling kit (Roche, USA). The transcribed probes were then incubated with the leaf sheath tissue sections. Finally, they were washed and incubated with an anti-digoxigenin-AP (200-052-156, Jackson ImmunoResearch Inc., PA, USA). The RNA hybridization signals were detected at room temperature by staining with a nitro-blue tetrazolium/5-bromo-4-chloro-30-indolyphosphate stock solution (NBT/BCIP solution; Boster Bio, CA, USA). Images were taken in the bright field mode using a microscope (Nikon Eclipse ci, Nikon Instruments Inc., NY, USA).

## Results

### Characteristics of the constructed single-cell transcriptome library of the leaf sheaths of BPH-resistant rice variety

**Figure 1:**
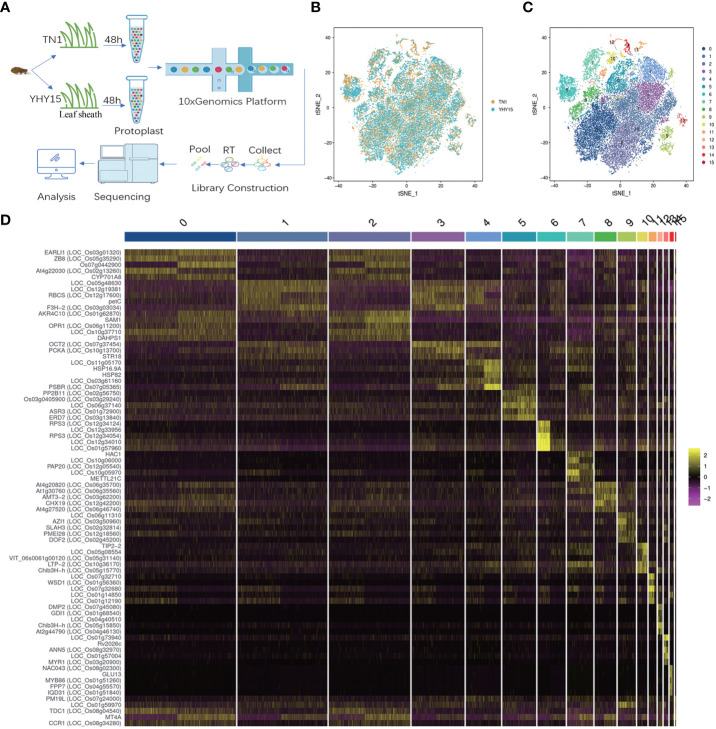
Quality control of the rice seedlings scRNA-seq analysis. **(A)** Representative schematic graph showing the workflow of scRNA-seq; **(B)** Representative plot of dimensional reduction of TN1 and YHY15; [Orange- TN1, Blue- YHY15] **(C)** Representative plot of the 16 major clusters of the TN1 and YHY15 cell transcriptomes; **(D)** Top 5 genes exhibiting upregulated expression in 16 clusters. [Yellow- high expression, Purple- low expression].

We isolated protoplasts from rice leaf sheaths after a 48 h infestation with the BPH to generate a single-cell transcriptome of the rice resistant to BPH. The 10x Genomics Chromium and the Illumina sequencing platforms were used to generate scRNA-Seq libraries (**Figure 1A**). For TN1 and YHY15 samples, we obtained 23,346 and 19,775 reads per cell, respectively. 1,670 expressed genes and 4,600 unique molecular identifiers (UMI) were generated for each cell. In TN1 and YHY15, we also detected 27,740 and 26,710 genes, respectively (**Supplementary Tables 1–3**). Using t-distributed stochastic neighbor embedding (tSNE) projection, the single-cell transcriptomes were plotted, and largely overlapping distributions between TN1 and YHY15 were observed, suggesting a high reproducibility (**Figure 1B**). To categorize the single cells, the package “Seurat” was applied. The results showed 16 major clusters of cell transcriptomes in two-dimensional space (**Figure 1C**). The total cells for TN1 and YHY15 were 14699 and 16237, respectively. Among these 16 clusters, the proportion of cells in most clusters was not significantly different, but the proportion of YHY15 cells was relatively low in clusters 11–14. In contrast, in cluster 15, the cell ratio of YHY15 was higher (**Supplementary Table 4**). Next, we examined the genes significantly upregulated in the 16 clusters, and the top 5 highly expressed genes were selected in each cluster (**Supplementary Table 5**); the expression pattern of these genes showed apparent cluster specificity (**Figure 1D**).

### Tissue-specific marker gene analysis of the BPH-inoculated rice leaf sheath transcriptome library revealed nine cell/tissue types

**Figure 2:**
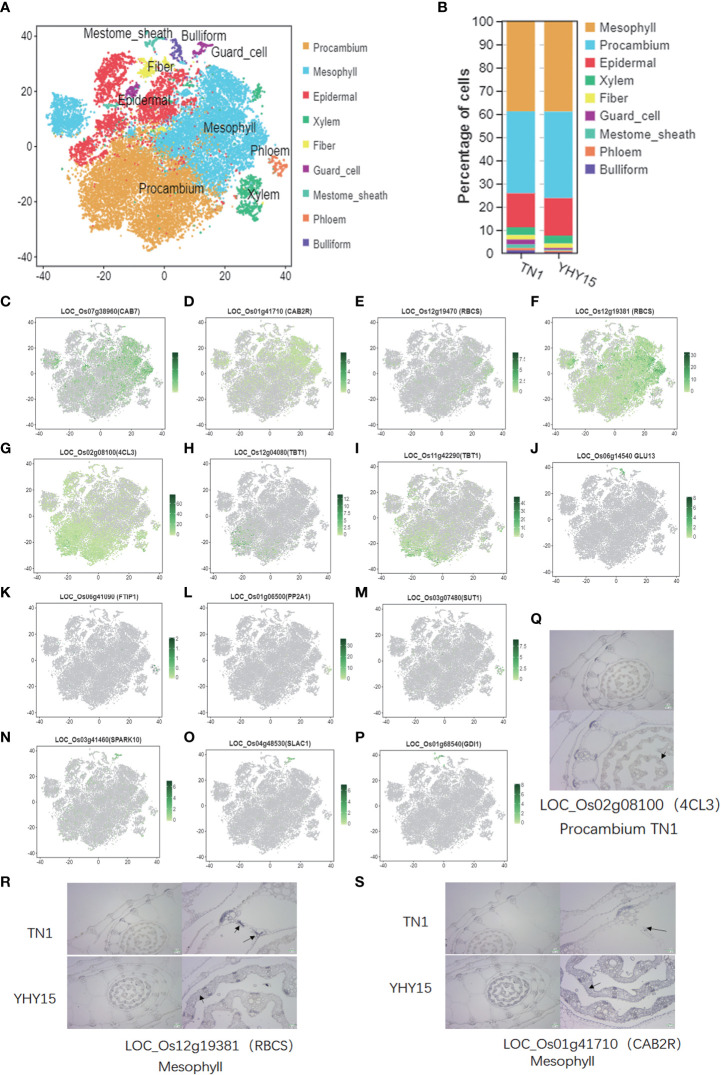
Single-cell transcriptome atlas for the rice leaf. **(A)** Representative plot of the combined accumulation of transcript from the tested marker genes (listed in **Supplemental Table S1**); **(B)** The percentage of cells in cell-type clusters; **(C–P)** tSNE plots of marker genes predicting the identities of clusters [The color scale indicates normalized expression level]; **(C–F)** Mesophyll maker genes, *LOC_Os07g38960* (*CAB7*), *LOC_Os01g41710* (*CAB2R*), *LOC_Os12g19470* (*RBCS*), and *LOC_Os12g19381* (*RBCS*); **(G–I)** Procambium maker genes, *LOC_Os02g08100* (*4CL3*), *LOC_Os12g04080* (*TBT1*), and *LOC_Os11g42290* (*TBT1*); **(J)** Bulliform maker gene, *LOC_Os06g14540* (*GLU13*); **(K–M)** Phloem maker genes, *LOC_Os06g41090* (*FTIP1*), *LOC_Os01g06500* (*PP2A1*), and *LOC_Os03g07480* (*SUT1*); **(N, O)** Guard cell maker genes, *LOC_Os03g41460* (*SPARK10*), *LOC_Os04g48530* (*SLAC1*); **(P)** Mestome sheath maker gene, *LOC_Os01g68540* (*GDI1*); **(Q–S)** Representative images showing the results of *in situ* hybridization. [Black triangles- the identified cell types and gene ID; the scale bare is shown in images].

For the assignment of tissue/cell type to clusters, the accumulated transcripts in our single-cell population were analyzed for significant expression of 52 marker genes in leaf sheath tissue/cell types (**Supplementary Table 6**). Nine major cell-type clusters were observed in both TN1 and YHY15 (**Figure 2A**) transcriptomes. Through comparing the changes in cell proportions for each cell type, we found that the proportions of mesophyll did not change much, but the proportions of procambium, guard cell, mestome sheath cell, and phloem were quite different (**Figure 2B**). The expression distribution of some existing marker genes, such as—1) Mesophyll marker genes (*LOC_Os07g38960* (*CAB7*), *LOC_Os01g41710* (*CAB2R*), *LOC_Os12g19470* (*RBCS*), and LOC_*Os12g19381* (*RBCS*)); 2) Procambium marker genes (*LOC_Os02g08100* (*4CL3*), *LOC_Os12g04080* (*TBT1*), and *LOC_Os11g42290* (*TBT1*)); 3) Bulliform marker genes (*LOC_Os06g14540* (*GLU13*)); 4) Phloem marker genes (*LOC_Os06g41090* (*FTIP1*), *LOC_Os01g06500* (*PP2A1*), and *LOC_Os03g07480* (*SUT1*)); 5) Guard cell marker genes (*LOC_Os03g41460* (*SPARK10*) and *LOC_Os04g48530* (*SLAC1*)); and 6) Mestome sheath marker genes (*LOC_Os01g68540* (*GDI1*))—were analyzed in each cell-type cluster. The expression of these genes was consistent with previously reported (**Figures 2C–P**). Therefore, we focused on detecting expression patterns of three selected genes, viz., *LOC_Os02g08100* (*4CL3*), *LOC_Os01g41710* (*CAB2R*), and *LOC_Os12g19381* (*RBCS*). The RNA *in situ* hybridization results confirmed the marker gene *LOC_Os02g08100* (*4CL3*) expression in procambium cells at the center of immature young leaf sheath (**Figure 2Q**). In mesophyll cells, a high RNA abundance of the mesophyll marker genes–*LOC_Os12g19381* (*RBCS*) and *LOC_Os01g41710* (*CAB2R*)–was seen, indicating that the annotation of cell types was reliable (**Figures 2R, S**).

### Functional enrichment of each cluster

**Figure 3:**
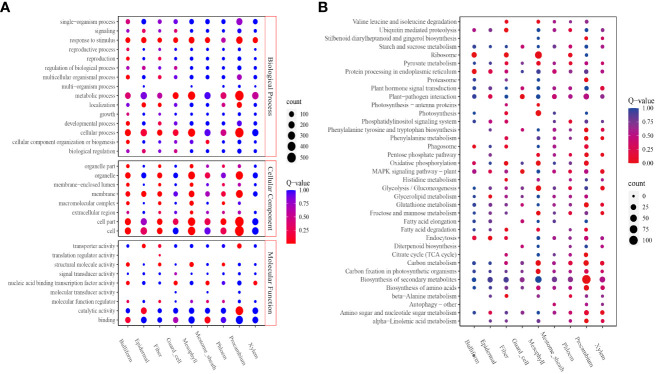
Function enrichment analyses. **(A)** GO analysis of all clusters; **(B)** KEGG analysis of all clusters. The level 2 GO terms, such as biological process, molecular function, cellular components, and the top 5 Go pathways and terms, are shown. The number of enriched genes was represented as the size of the circle. The significant enrichments were presented in red, and the insignificant enrichments were presented in blue.

The DEGs upregulated in each cluster were identified to obtain the cell types’ basic information in all clusters; 3,780 upregulated genes were screened (**Supplementary Tables 7, 8**). In all, 208 to 856 DEGs were identified. Procambium cells, followed by mesophyll and epidermal cells, had the highest number of DEGs; the least number of DEGs were found in fiber cells (**Supplementary Table 7**). Next, the potential enriched pathways and functions were determined by KEGG and GO analyses. GO analysis revealed that all eight cell-type clusters (except the phloem cluster) were significantly enriched in the “response to stimulus” biological process. The guard cell, mesophyll, and procambium clusters were significantly enriched in the “metabolic process.” Bulliform, epidermal, fiber, guard cell, mesophyll, and procambium clusters were significantly enriched in the “cellular process” (**Figure 3A**). The molecular function results showed bulliform and mestome sheath were significantly enriched in the “binding” terms. Epidermal and procambium clusters were significantly enriched in “catalytic activity” and “transporter activity” (**Figure 3A**).

KEGG analysis revealed that the bulliform cluster was significantly enriched in “ribosome,” “protein processing in the endoplasmic reticulum,” and “phagosome” pathways. The epidermal cluster was significantly enriched in “endocytosis,” “glycerolipid-,” and “glycerophospholipid-” metabolism pathways. Fiber cluster was significantly enriched in “ubiquitin-mediated proteolysis,” “ribosome,” “oxidative phosphorylation,” “β-Alanine metabolism,” “fatty acid degradation,” “valine, leucine, and isoleucine degradation,” and “histidine metabolism” pathways. Guard cell cluster was significantly enriched in “plant-pathogen interaction” and “MAPK signaling” pathways. Mesophyll cluster was significantly enriched in “ribosome,” “photosynthesis,” “carbon fixation in photosynthetic organisms,” “glycolysis/gluconeogenesis,” “oxidative phosphorylation,” “carbon metabolism,” “mannose and fructose metabolism,” ‘photosynthesis-antenna proteins,” and “valine, leucine, and isoleucine degradation” pathways. Procambium cluster was significantly enriched in “citrate cycle (TCA cycle),” “phenylalanine metabolism,” “proteasome,” “tryptophan, tyrosine, and phenylalanine biosynthesis,” “α-linolenic acid metabolism,” “biosynthesis of amino acids,” “carbon metabolism,” “biosynthesis of secondary metabolites,” “glutathione metabolism,” “pentose phosphate pathway,” “oxidative phosphorylation,” and “stilbenoid, diarylheptanoid, and gingerol biosynthesis” pathways (**Figure 3B**).

The bulliform cluster was similar to the mestome sheath, and the epidermal cluster was similar to the procambium in the Go enrichment profiles.

### Susceptible and resistant rice varieties mounted different responses to BPH feeding, which also differed based on cell type

**Figure 4:**
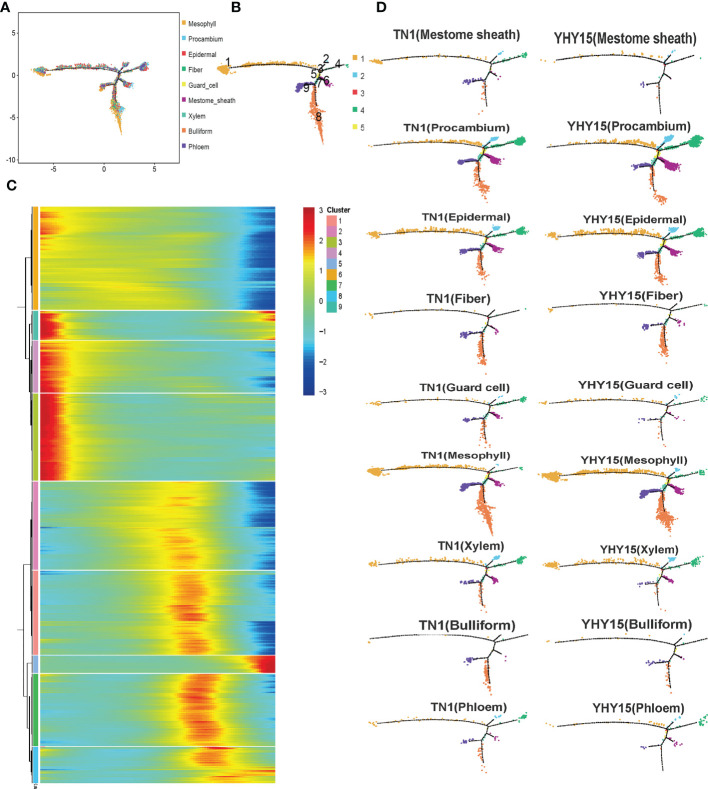
Pseudotime trajectory of all cluster’s cells. **(A)** Pseudotime analysis using Monocle for cell transcriptomes; **(B)** The state information of differentiation; **(C)** Gene expression heatmap for all cluster genes; **(D)** Expression profile in differentiation state of all subgroup cells.

The pseudotime trajectory analysis of all cell-type clusters was conducted to evaluate the responses of the rice cells to BPH infestation (**Figure 4A**). The development and response processes to BPH feeding could be divided into nine differentiation states (states 1–9) (**Figure 4B**). Pseudotime path clustering of DEGs revealed branching in the gene expression pattern of the differentiation states (**Figure 4C**, **Supplementary Table 9**). Susceptible and resistant rice varieties can mount different gene expression responses to BPH infestation. By comparing the differences in each cell type in TN1 and YHY15, we found that the procambium, epidermal, and fiber cells are similar across all differentiation states. Between TN1 and YHY15, significant differences in differentiation states were observed as follows–a) mestome sheath cells-states 1, 4, 6, and 8; b) guard cells-states 4, 6, 8, 9; c) mesophyll cells-state 8; d) xylem cells-states 1, 6, 8, 9; e) bulliform cells-states 8, 9; and f) phloem cells-states 1, 4, 6, 8, 9 (**Figure 4D**).

### Expression of genes related to vanillin, capsaicin, and ROS production in the mesophyll cluster depends on BPH-susceptibility

**Figure 5:**
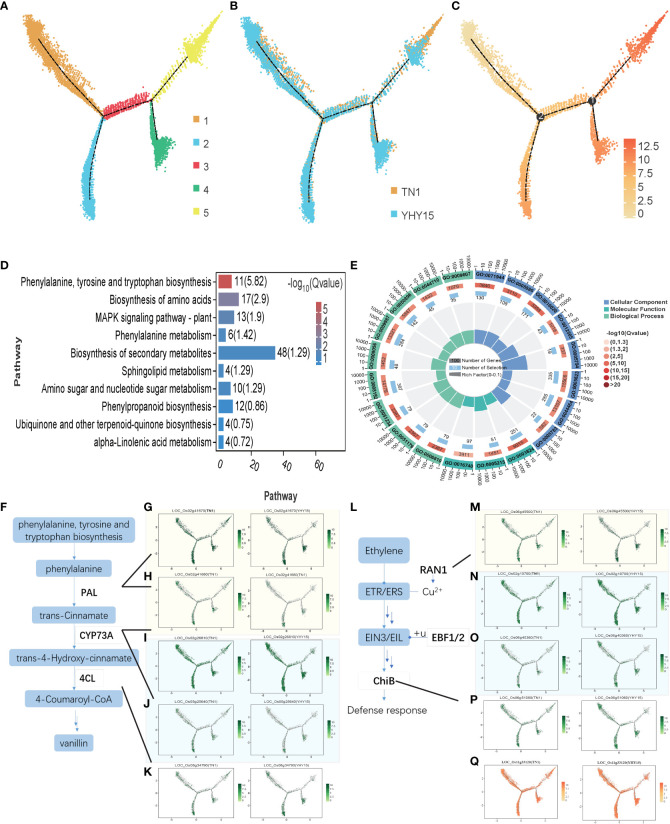
Pseudotime trajectory and function analysis of Mesophyll cells. **(A)** The state information of Mesophyll cells differentiation; **(B)** Samples information of Mesophyll cells; **(C)** Pseudotime trajectory of Mesophyll cells; **(D)** KEGG enrichment analysis of cluster 5 of Mesophyll cells; **(E)** GO enrichment analysis of cluster 5 of Mesophyll cells; **(F)** The schematic diagram of phenylalanine pathway; **(G–K)** The expression profiles of genes *LOC_Os02g41670* (*PAL*) **(G)**; *LOC_Os02g41680* (*PAL*) **(H)**; *LOC_Os02g26810* (*CYP73A*) **(I)**; *LOC_Os05g25640* (*CYP73A*) **(J)**, and *LOC_Os08g34790* (*4CL*) **(K)** in TN1 and YHY15 samples; **(L)** The schematic diagram of ethylene response pathway; **(M–Q)** The expression profiles of genes *LOC_Os06g45500* (*RAN1*) **(M)**; *LOC_Os02g10700* (*EBF1/2*) **(N)**; *LOC_Os06g40360* (*EBF1/2*) **(O)**; *LOC_Os06g51060* (*ChiB*) **(P)** and *LOC_Os11g33120* (*ChiB*) **(Q)** in TN1 and YHY15 samples.

Although epidermal cells are the primary barrier of plants when BPH suck the phloem sap, their stylet bundle mainly pierces mesophyll cells; thus, mesophyll cells form a secondary barrier against further BPH feeding. The pseudotime trajectory analysis results of the mesophyll cells showed that the mesophyll cells were mainly divided into five states (**Figure 5A**). Comparing the gene expression in TN1 and YHY15 showed that the differences were primarily concentrated in branch 5 (**Figure 5B**). The overall expression level in cluster 5 was increased compared to that in other branches (**Figure 5C**). KEGG enrichment analysis of cluster 5 showed that these genes were significantly enriched in “phenylalanine, tyrosine, and tryptophan biosynthesis,” “biosynthesis of amino acid,” “MAPK signaling,” and “phenylalanine metabolism” pathways (**Figure 5D**, **Supplementary Table 10**). Phenylalanine metabolism is one of the downstream regulatory pathways of phenylalanine, tyrosine, and tryptophan biosynthesis. In this pathway, the phenylalanine ammonia-lyase (PAL) gene expression–viz. (*LOC_Os02g41670*, *LOC_Os02g41680*), *CYP73A* (*LOC_Os02g26810*, *LOC_Os05g25640*) and *4CL* (*LOC_Os08g34790*)–were mainly affected. These are part of gene regulatory pathways that affect the 4-Coumaroyl-CoA levels and, consequently, the levels of vanillin or capsaicin. The GO enrichment analysis of cluster 5 genes showed that the main enriched GO terms include “response to stimulus” (GO: 0050896), “response to biotic stimulus” (GO: 0009607), etc. (**Figure 5E**). Three genes–two PAL homologs, two CYP73A homologs, and a 4CL homolog (**Figures 5G–K**)–in the phenylalanine and vanillin synthesis pathways were significantly different between the two rice varieties (**Figure 5F**). Further, cell number and expression of five genes in cluster 5 were quite different between TN1 and YHY15 varieties. Furthermore, the ethylene response pathway genes–*RAN1*, *EBF1/2*, and *ChiB*–under the MAPK signaling pathway had significant differences in expression levels (**Figures 5L–P**); thus, they may be involved in the trauma response of rice to BPH feeding. In addition, the expression of cluster 5 gene *LOC_Os11g33120* (encoding a *ROBH* gene involved in reactive oxygen species (ROS) production) was significantly higher in TN1 than in YHY15. Thus, BPH feeding may have triggered differences in ROS production (**Figure 5Q**).

### Phloem cell

**Figure 6:**
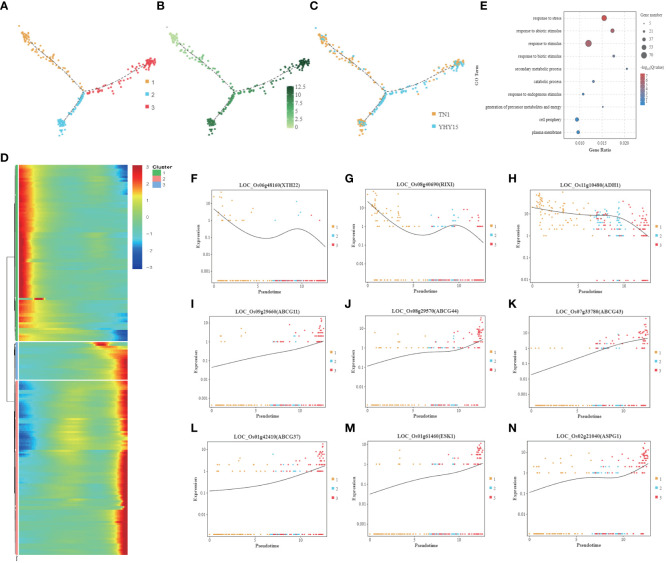
Pseudotime trajectory and function analysis of Phloem cells. **(A)** Pseudotime trajectory of Phloem cells; **(B)** The state information of Phloem cells differentiation; **(C)** Samples information of Phloem cells; **(D)** Gene expression heatmap for all cluster genes of Phloem cells; **(E)** GO enrichment analysis of Phloem cells; **(F–N)** Representative graph showing the trend of the selected DEGs expression along pseudotime trajectory during differentiation for each cell type- **(F)** *LOC_Os06g48160* (*XTH22*); **(G)** *LOC_Os08g40690* (*RIXI*); **(H)** *LOC_Os11g10480* (*ADH1*); **(I)** *LOC_Os09g29660* (*ABCG11*); **(J)** *LOC_Os08g29570* (*ABCG44*); **(K)** *LOC_Os07g33780* (*ABCG43*); **(L)** *LOC_Os01g42410* (*ABCG37*); **(M)** *LOC_Os01g261460* (*ESK1*); **(N)** *LOC_Os08g21040* (*ASPG1*). [One single cell was represented as one point. The entire X-axis was defined as “Pseudotime,” entire Y-axis was defined as “Relative expression”].

Since BPH sucks phloem sap, the feedback of phloem cells to stress is inextricably linked to rice BPH resistance. The pseudotime trajectory analysis showed that the phloem cells were mainly divided into three clusters (**Figure 6A**). The gene expression level was lowest in cluster 1 and up-regulated in clusters 2 and 3 (**Figure 6B**). Cluster 1 mainly consists of TN1 cells, while clusters 2 and 3 have more YHY15 cells (**Figure 6C**). The gene expression level of cluster 1 gradually decreased along pseudotime, while clusters 2 and 3 showed an increasing trend (**Figure 6D**). The main GO enrichment terms for these genes include “response to stress,” “response to abiotic stimulus,” and “response to stimulus” (**Figure 6E**). Cluster 1 has multiple genes closely related to cell wall extension, such as *LOC_Os06g48160* (*XTH22*, xyloglucan endotransglucosylase/hydrolase protein 22) (**Figure 6F**), *LOC_Os08g40690* (*RIXI*, xylanase inhibitor protein 1) (**Figure 6G**). It also has energy production genes *LOC_Os11g10480* (*ADH1*, alcohol dehydrogenase I) (**Figure 6H**). Multiple ABC transporter G family members in cluster 3–such as *LOC_Os09g29660* (*ABCG11*) (**Figure 6I**), *LOC_Os08g29570* (*ABCG44*) (**Figure 6J**), *LOC_Os07g33780* (*ABCG43*) (**Figure 6K**), *LOC_Os01g42410* (*ABCG37*) (**Figure 6L**), *LOC_Os01g261460* (*ESK1*, promotes xylan acetylation) (**Figure 6M**), *LOC_Os08g21040* (*ASPG1*, aspartic protease in guard cell) (**Figure 6N**)–exhibited significantly elevated expression.

### Xylem cells

**Figure 7:**
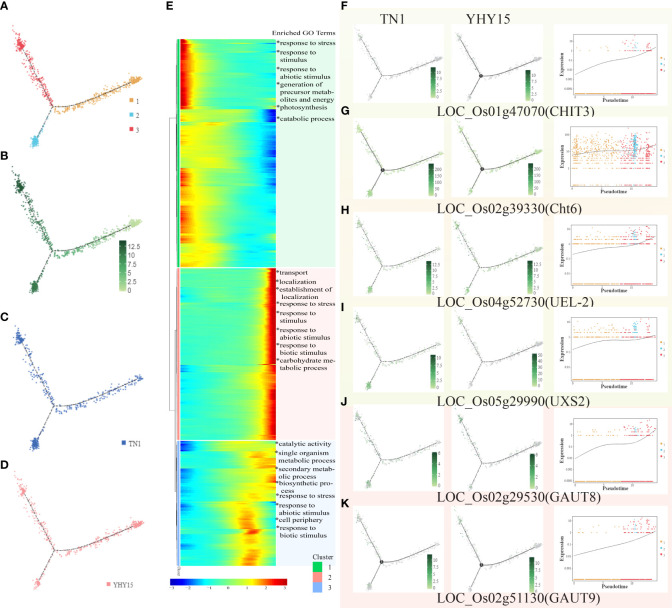
Pseudotime trajectory and function analysis of Xylem cells. **(A)** The state information of Xylem cells differentiation; **(B)** Pseudotime trajectory of Xylem cells; **(C)** The expression pattern of Xylem cells in TN1; **(D)** The expression pattern of Xylem cells in YHY15; **(E)** Gene expression heatmap and GO enrichment analysis for all cluster genes of Xylem cells; **(F–K)** Representative graphs showing the profiles of the selected DEGs expression and trend- **(F)** *LOC_Os01g47070* (*CHIT3*); **(G)** *LOC_Os02g39330* (*Cht6*); **(H)** *LOC_Os04g562730* (*UEL-2*); **(I)** *LOC_Os05g29990* (*UXS2*); **(J)** *LOC_Os02g29530* (*GAUT8*); **(K)** *LOC_Os02g51130* (*GAUT9*). [One single cell was represented as one point. The entire X-axis was defined as “Pseudotime,” entire Y-axis was defined as “Relative expression”].

BPH also sucks xylem sap (Seo et al., 2009). The xylem cells were divided into 3 clusters (**Figure 7A**). The overall expression level was lowest in cluster 1 and higher in clusters 2 and 3 (**Figure 7B**). Among the three clusters, the main difference between the two varieties was found in cluster 2 (**Figures 7C, D**). The gene expression level in cluster 1 gradually decreased; it was enriched in genes associated with GO terms “response to stress.” On the other hand, the gene expression level in clusters 2 and 3 showed a gradual increase; they were enriched in genes associated with GO terms, including “transport,” “localization,” “response to stress,” etc. Cluster 3 was enriched with genes associated with GO terms, such as “catalytic activity,” “secondary metabolic process,” “biosynthetic process,” and “response to stress” (**Figure 7E**). However, enrichment of KEGG pathways for the three clusters showed that cluster 1 was mainly enriched in pathways associated with “glycolysis/gluconeogenesis,” cluster 2 was enriched primarily in pathways such as “phenylalanine, tyrosine, and tryptophan biosynthesis,” including “amino sugar and nucleotide sugar metabolism,” especially chitin related genes, such as *LOC_Os01g47070* (*CHIT3*), *LOC_Os02g39330* (*Cht6*), *LOC_Os04g52730* (*UEL-2*), *LOC_Os05g29990* (*UXS2*), and so on. In addition, some pectin-related genes (such as *LOC_Os02g29530* (*GAUT8*), *LOC_Os02g51130* (*GAUT9*), etc.) showed significant changes (**Figures 7F–K**). The major KEGG pathways enriched in cluster 3 are “phenylpropanoid biosynthesis,” “phenylalanine metabolism,” and “phenylalanine, tyrosine, and tryptophan biosynthesis.”

## Discussion

BPH is one of the most damaging rice pests, causing substantial economic losses. Breeding BPH-resistant rice varieties has been a successful strategy, but BPH can eventually co-evolve and adapt to the resistance mechanisms of rice plants. BPH has a stylet bundle mouthpart that can pierce and suck the phloem sap of rice. However, when feeding on resistant rice varieties, BPH may fail to reach the phloem and stop feeding due to the presence of repellent substances in any cell type along the way. Therefore, analyzing the BPH-feeding stimulated cell expression patterns of different rice plant tissues will help identify the cells that mediate rice resistance to BPH and reveal the underlying molecular mechanisms. Through enabling transcriptomic analysis at single-cell resolution, the application of scRNA-seq has significantly revolutionized the study of cell and molecular biology. Moreover, it has dramatically enhanced our ability to characterize cell states and gene expression responses to BPH feeding. Here, we first constructed a single-cell atlas of rice leaf sheath response to BPH infestation.

Previous studies have used scRNA-seq technology to investigate tissue and organ development and differentiation in rice, thus, identifying marker genes that served as important references for our research (Xu et al., 2021b; Zhang et al., 2021a; Zong et al., 2022). However, reports on how different cell types respond–either by producing resistance chemicals or activating other molecular processes –to BPH feeding are lacking. Therefore, we selected 48 h post-infestation time for this study based on previous results that show significant changes in gene expression occurring between 24–48 hours of BPH infestation of rice leaves (Liu Y. et al., 2021; Xu et al., 2021a; Xue et al., 2023).

Here, we found that mainly mestome sheath cells, guard cells, mesophyll cells, xylem cells, bulliform cells, and phloem cells showed differential gene expression in resistant (YHY15) and susceptible (TN1) rice varieties after BPH infestation (**Figure 4**). This suggests that the factors imparting resistance to BPH may originate from multiple sources; different cells may contribute to insect resistance. Thus, the combined effects of numerous resistance factors may confer insect-resistance properties on plants. Previous studies using electrical penetration graphs and honeydew clocks have shown that rice resistance to BPH is determined by differences in sustained phloem ingestion, not by phloem location (Ghaffar et al., 2011). However, in the susceptible TN1 variety, BPH ingests phloem sap continuously without interruption (Ghaffar et al., 2011). Our results further support that rice resistance to BPH does not arise from a single resistance factor in the phloem but from a combination of resistance factors present in multiple locations in the rice plant.

Here, we found that the cell numbers were higher for mesophyll, procambium, and epidermal cells, with a significant difference in the proportion of mesophyll cells (cluster 4 in **Figure 1**, **Supplementary Table 4**) and procambium cells (cluster 2 in **Figure 1**, **Supplementary Table 4**). In contrast, the proportion of epidermal cells was less different in TN1 and YHY15 rice varieties (clusters 5, 7, and 8 in **Figure 1**, **Supplementary Table 4**). In addition, although the proportion of guard, mestome sheath, and bulliform cells differed approximately by 1% between TN1 and YHY15 rice varieties, the number of these cell types was relatively small. Moreover, procambium cells are the precursors of differentiated mature cells. Further, BPH feeding may mainly involve xylem and phloem sap ingestion (Sōgawa, 1982; Seo et al., 2009). Therefore, we focused on mesophyll, xylem, and phloem cells in this study.

Basal resistance is present in both susceptible and resistant rice varieties. The release of green leaf volatiles, which can prevent BPH infestation, is promoted by BPH feeding (Qi et al., 2011). Additionally, MAPKs, ethylene, and salicylic acid (SA) related signaling pathways were also activated by BPH feeding (Du et al., 2009; Hu et al., 2011; Lu et al., 2011). Here, the DEGs of mesophyll cells in TN1 and YHY15 varieties were mainly related to “phenylalanine, tyrosine, and tryptophan biosynthesis,” “MAPK signaling pathway,” and “phenylalanine metabolism.” PALs is a crucial enzyme that mediates the resistance to BPH by regulating the biosynthesis and accumulation of SA and lignin (He et al., 2020). We found that the downstream genes of the PAL pathway (*CYP73A* and *4CL*), which may mainly induce the synthesis of vanillin and other compounds, were significantly differentially expressed in the two varieties. Vanillin-containing plants have vigorous insecticidal and insect-repellent activities (Kim et al., 2012; Songkro et al., 2012; Kletskova et al., 2017). MAPK signaling pathway is an essential part of the ethylene signaling transduction, which also plays a crucial role in plant defense response to insects (Hu et al., 2011; Hettenhausen et al., 2015; Zhou et al., 2019). The copper-transporting ATPase RAN1 is essential for the biogenesis of ethylene receptors (Binder et al., 2010). F-box proteins EBF1/EBF2 can form SCF complex to degrade EIN3 protein and regulate the expression level of downstream gene *ChiB* which is involved in the rice defense response to BPH (Zhu and Guo, 2008).

In phloem cells, the functions associated with DEGs in the two varieties mainly include cell wall extension, energy production, etc. However, cells of the susceptible variety TN1 were mainly concentrated in cluster 1, primarily associated with cell wall extension function. While clusters 2 and 3 had more cells of the BPH-resistant YHY15 variety, whose function was mainly related to “response to biotic stimulus” (**Figure 6**). Clusters 2 and 3 were also enriched in “phenylpropanoid biosynthesis pathway genes” such as *LOC_Os02g41670* (*PAL*), *LOC_Os05g35290* (*PAL*), *LOC_Os08g34790* (*4CL5*), *LOC_Os02g08100* (*4CL3*), *LOC_Os09g04050* (*CCR1*), and *LOC_Os01g73200* (*PRDX6*); these genes may promote lignin biosynthesis.

In xylem cells, we found that although DEGs were mainly enriched with genes significantly associated with stress response, their mainly enriched KEGG pathways were practically different, especially cluster 2 (**Figure 7**), which contained multiple genes related to chitin and pectin metabolism. In plants, chitin oligosaccharides induce various defense responses across multiple plant cells (Kaku et al., 2006). Pectin is a critical component of the cell wall. Therefore, pectin metabolism may be crucial in cell wall integrity and mediate plant defense responses (Wang et al., 2023).

In summary, the differences in immune responses stimulated by BPH infestation–such as MAPK signaling pathway and lignin biosynthesis for preventing callose and cell wall degradations that restrict BPH feeding and disrupt BPH digestion–are caused by the existing differences in TN1 and YHY15 varieties. Compared to the susceptible rice variety, the amplified and accelerated responses of the resistant rice variety may protect the plant from further BPH attack, allowing them to survive. The results of scRNA-seq suggest that multiple resistive factors may work together to make rice plants resistant to BPH, and different cell types may have other molecular mechanisms underlying BPH resistance.

In this study, we successfully—1) mapped a single cell transcriptome atlas of rice leaf sheath affected by BPH infestation; 2) observed the relationship of differentiation among the cell clusters; and 3) identified multiple cell types that may be involved in BPH-defense response mounted by rice. However, further experimental evidence is needed to elucidate the role of multiple cells and genes in BPH resistance. Nevertheless, our investigation provides a basis for mining insect-resistance genes and deciphering the molecular mechanism underlying insect-resistance and importantly guides insect-resistant plant molecular breeding.

## Data availability statement

The raw sequence data reported in this paper have been deposited in the Genome Sequence Archive in National Genomics Data Center (https://ngdc.cncb.ac.cn/), China National Center for Bioinformation/Beijing Institute of Genomics, Chinese Academy of Sciences, under project number: PRJCA014345, accession number CRA009555 that are publicly accessible at https://ngdc.cncb.ac.cn/gsa.

## Author contributions

WZ, DX, LZ, and AY conceived and designed the experiments. WZ performed most of the experiments. CL, JC, and YW analyzed the data, authored or reviewed article drafts, and approved the final draft. MS analyzed the data. SL and BW provided help with the Q16 RNA *in situ* hybridization experiments. SS, KL, and HX prepared figures and/or tables. PL and KL helped to collect the samples. Finally, GY and ZC helped to revise the manuscript. All authors contributed to the article and approved the submitted version.
